# Feasibility and initial efficacy of a culturally sensitive women-centered substance use intervention in Georgia: Sex risk outcomes

**DOI:** 10.1186/s13011-015-0043-0

**Published:** 2015-12-08

**Authors:** Hendrée E. Jones, Irma Kirtadze, David Otiashvili, Keryn Murphy, Kevin E. O’Grady, William Zule, Evgeny Krupitsky, Wendee M. Wechsberg

**Affiliations:** UNC Horizons, Department of Obstetrics and Gynecology, School of Medicine, University of North Carolina at Chapel Hill, 127 Kingston Drive, Chapel Hill, NC 27514 USA; Departments of Psychiatry and Behavioral Sciences and Obstetrics and Gynecology, School of Medicine, Johns Hopkins University, Baltimore, MD 21224 USA; Addiction Research Center, Alternative Georgia, Tbilisi, 0177 Georgia; Ilia State University, Business School, Tbilisi, 0162 Georgia; Department of Psychology, University of Maryland, College Park, College Park, MD 20742 USA; Substance Abuse Treatment Evaluations and Interventions Research Program, RTI International, Research Triangle Park, ᅟ, NC 27709 USA; Department of Addictions, Bekhterev Research Psychoneurological Institute, St. Petersburg, 192019 Russia

**Keywords:** Women-centered treatment, Injection drug use, Georgia, HIV prevention, Risky sexual behavior

## Abstract

**Background:**

This paper reports on the feasibility and initial efficacy of a culturally sensitive, comprehensive women-centered substance use intervention for women who inject drugs in Georgia in terms of the primary and secondary sex risk outcomes. The hypothesis under examination was that, relative to case management participants, participants in a culturally sensitive, comprehensive women-specific and -centered intervention would, on average, show significant decreases in past-30-day frequency of unprotected sex, unprotected sex at the last sexual encounter, and increases in condom use and safer sex actions.

**Methods:**

The study was a two-arm randomized trial, in which 173 potentially eligible women were screened, and those 128 women determined to be eligible were assigned at random to either Reinforcement-based Treatment plus Women’s Co-Op (RBT + WC) or case management (CM). RBT + WC participants received 12 sessions of a structured intervention with the goal of reducing risky sex and substance use and improving physical and mental health. CM participants received 12 sessions of case management and informational brochures that focused on the same issues on which RBT + WC focused. Participants were assessed at baseline, post-treatment, and 3 months following treatment enrollment.

**Results:**

Analyses revealed case management having significantly overall higher Safer Sex action scores than RBT + WC, and a significant decrease over time for past 30-day number of unprotected sex acts. Unprotected sex at the last encounter and Condom Use action scores were nonsignificant.

**Conclusions:**

Women who inject drugs in Georgia are engaging in risky sexual practices, and are in need of an intervention that addresses these risky behaviors. Reasons for the failure to find differences between a culturally sensitive, comprehensive women-centered intervention and case management tailored to the needs of women who inject drugs in Georgia may have been the result of inadequate power to detect an effect in a sample whose drug use was not as serious as warranted by the intervention. (ClinicalTrials.gov Identifier: NCT01331460)

## Background

Substance use disorders are a worldwide problem [1]. According to the World Health Organization “Opiates and opioids top the list of problem drugs that cause the most burden of disease and drug-related deaths worldwide” [1]. International guidelines exist for the treatment of opioid use disorder, [2] although the United States has witnessed the preponderant amount of research related to the treatment of opioid use disorder. Moreover, although the World Health Organization has focused attention on the treatment of pregnant women with substance use disorders [3], little attention has been paid to the development of women-centered treatment for substance use disorders in Georgia, despite documentation of the unique needs of women in substance use treatment [4], and research in the US that has shown that treatments that focus on the issues more commonly found in women with substance use disorders may be more efficacious [5–7]. Despite the confluence of findings in this regard in the US, and the increasing emphasis on women’s issues by the US Substance Abuse and Mental Health Services Administration (for example, *Women Matter *[8]), international research that has focused on the development and validation of women-centered treatments for women with substance use disorders has not been widespread, although there have been substantive efforts to develop such treatments [9–14]. Of particular note in regard to the present paper is the development of a comprehensive, women-centered intervention for women who inject drugs in Georgia [15].

Women who inject drugs in Georgia encounter continuing stigma and discrimination. Women have few opportunities to receive publicly funded substance use treatment services in Georgia. Women represent approximately 10 % of the substance-using adult population [16, 17], yet only 1-5 % of the beneficiaries of substance use treatment services are women [18, 19]. If available, substance use treatment services in Georgia have been designed to serve male beneficiaries, and fail to address the unique needs and challenges that women who inject drugs face in their daily lives. Our own research [20] found that health service providers to substance-using women believed that drug dependence in women is much more severe than in men. Most providers believed that substance-using women were failures as mother, wife, and child. Moreover, substance-using Georgian women often report that that there are no available treatment programs that would address the specific needs of women [21].

Moreover, women who inject drugs in Georgia frequently experience emotional abuse, and physical and sexual abuse and violence [21]. These behaviors on the part of their husbands and sexual partners reflect a cultural and social environment that engenders male dominance and places restrictions on female equality and independence [22]. Moreover, women have been able to experience a broader range of social roles, and an attendant increase in employment opportunities, given the recent economic distress in Georgia. These changing sex roles have often lead to men attempting to reassert their positions of power and dominance through the use of physical force [23, 24], leading to heightened risk of abuse, particularly sexual abuse, given the attendant increased risk of HIV and HCV transmission from infected partners, due to inability to negotiate safe sex practices out of fear of abuse [21].

Not surprisingly, injection drug use among adults in Georgia has been associated with HIV infection [25], with women comprising 26.5 % out of the total number of registered cases. Among the 4930 HIV-positive cases registered with the National AIDS Centre by April 2015, 48.7 % were identified as injection-drug-using individuals [26]. Although the overall HIV prevalence in Georgia is low, the number of HIV-positive individuals registered with the National AIDS Centre rose at least 5–10 % annually between 1994 and 2009 [27], adding to the concern regarding an HIV epidemic in Georgia [28].

As in Western countries, injection drug use has also been closely tied with hepatitis C virus (HCV) infection. HCV seroprevalence among injection-drug-using adults in Georgia has been estimated to be 50-70 % [25, 29]. Although the HCV seroprevalence rate among injection-drug using adults in Tbilisi, capital city of Georgia was recently found to be significantly lower in women than in men [30], the rates for both men (95.0 %) and women (58.8 %) were alarmingly high.

The focus of the IMEDI (Investigating Methods for Enhancing Development in Individuals: IMEDI is the Georgian word for hope) project, funded by the US National Institute on Drug Abuse (R01 DA029880) in July, 2010, was on the development of a women-specific and -centered intervention with the objective of reducing HIV and HCV in women who inject drugs. IMEDI had two goals. The first goal was to gain the necessary knowledge from women who inject drugs and treatment providers about drug use, HIV risk behaviors, and the current drug treatment in Georgia. The second goal was to use this information to adapt, integrate, and implement a comprehensive treatment program that combines two efficacious interventions to slow the transmission of HIV and HCV in Georgia.

**Fig. 1:**
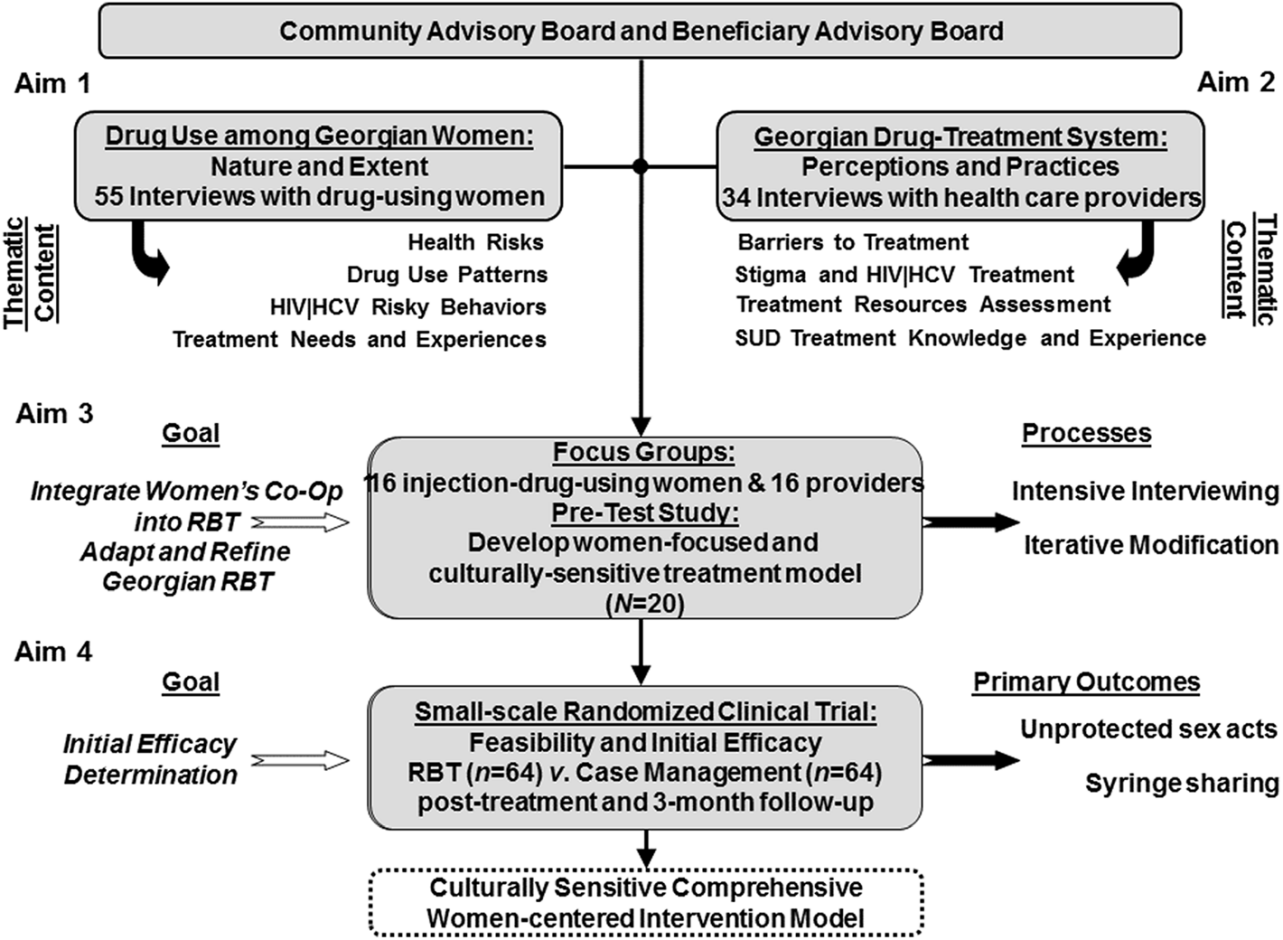
Overall design of the IMEDI project. Figure 1 in Jones HE, Kirtadze I, Otiashvili D, O’Grady KE, Murphy K, Zule W, Krupitsky E, Wechsberg WM. Process and product in cross-cultural treatment research: development of a culturally sensitive women-centered substance use intervention in Georgia. *J Addict* Sep 2014;Article ID 163603 © 2104 by Hendrée E. Jones et al. Used with permission

In order to address its twin goals, four different IMEDI studies focused on four separate aims (see Fig. 1). The first two studies involved collection and analysis of data from interviews with women who inject drugs [21], and with health care providers [20] who provide services to these women. These first two studies provided data to guide the development of a culturally sensitive, comprehensive, women-centered intervention for women who inject drugs. The aim of the third study [15] was the cyclical refinement and adaption of this intervention. The aim of the fourth study was to examine the feasibility and initial efficacy of the experimental intervention.

This paper reports on the feasibility and initial efficacy of our culturally sensitive, comprehensive, women-centered intervention in terms of the primary and secondary sex risk outcomes. The hypothesis under examination was that, relative to case management participants, participants in the culturally sensitive, comprehensive women-specific and -centered intervention would, on average, show significant decreases in past-30-day frequency of unprotected sex, unprotected sex at the last sexual encounter, and increases in condom use and safer sex actions.

## Methods

This study was approved by the Office of Human Research Ethics Institutional Review Board (IRB) at the University of North Carolina at Chapel Hill, USA and the IRB at the Maternal and Child Care Union, Georgia. All participants provided written informed consent at study entry.

### Study design

The study was a two-arm randomized trial. Women who met eligibility criteria and provided informed consent were administered a baseline assessment. After completion of the baseline assessment, they were randomized into either the experimental or control intervention condition. Women in both arms were offered two sessions a week for 6 weeks. Follow-up assessments were scheduled immediately after treatment completion and again 3 months following enrollment.

### Study site

An office suite with sufficient space for treatment and research was rented in the Saburtalo district of Tbilisi. This location was chosen because it had no previous association with substance use treatment services, in order to minimize barriers for participants to enter and complete the intervention.

### Recruitment

The IMEDI project had both a Community Advisory Board (CAB) and a Beneficiary Advisory Board (BAB). The CAB consisted of 11 members with expertise in health of and services to injection-drug-using women, including substance use treatment providers, professionals working in prisons and law enforcement, women’s violence intervention, providers of family planning and other aspects of women’s reproductive health, counselors that provide HIV and STI counseling, HIV prevention services, women in the Global Fund’s Country Coordinating Mechanism who oversee HIV and tuberculosis prevention and treatment programs in Georgia, and the director of a methadone maintenance program. The members of the BAB were 4 injection-drug-using women. The CAB and BAB provided input into all phases of the project. Based on their input, a number of sites in Tbilisi and Gori that substance-using women were known to frequent were identified.

Outreach workers used venue-based sampling methods to recruit participants at the identified sites. Venue-based sampling [31] is a time-space sampling method that involves identifying the days and times members of the population of interest assemble at a particular location, and randomly selecting members of the population present for screening or participation.

In the present study, consistent with venue-based sampling, sampling methods were adapted to fit the conditions at each site. Outreach workers were trained in the recruitment procedures and protocols and the recruitment procedure was scripted in a manual. They used standardized street outreach techniques to recruit injection-drug-using women at each site. A field screening instrument was used to make the initial determination of eligibility and refer potential participants to the study field office for the final determination. Eligibility criteria were: conversant in Georgian; minimum 18 years of age; able to provide informed consent; injection of illicit drugs in the past 30 days as verified by venipuncture stigmata; and self-report of heterosexual activity at least once in the past 30 days.

A research assistant at the field office who met with the potential participation and determined that she was eligible for study participant opened an opaque sealed envelope that indicated assignment to treatment condition. Envelopes were prepared by non-clinical project staff who had no participant contact using a block randomization procedure to assign each successive pair of participants to each of the treatment conditions.

No participant in this study had enrolled in a previous IMEDI project study.

### Treatment conditions

**Fig. 2:**
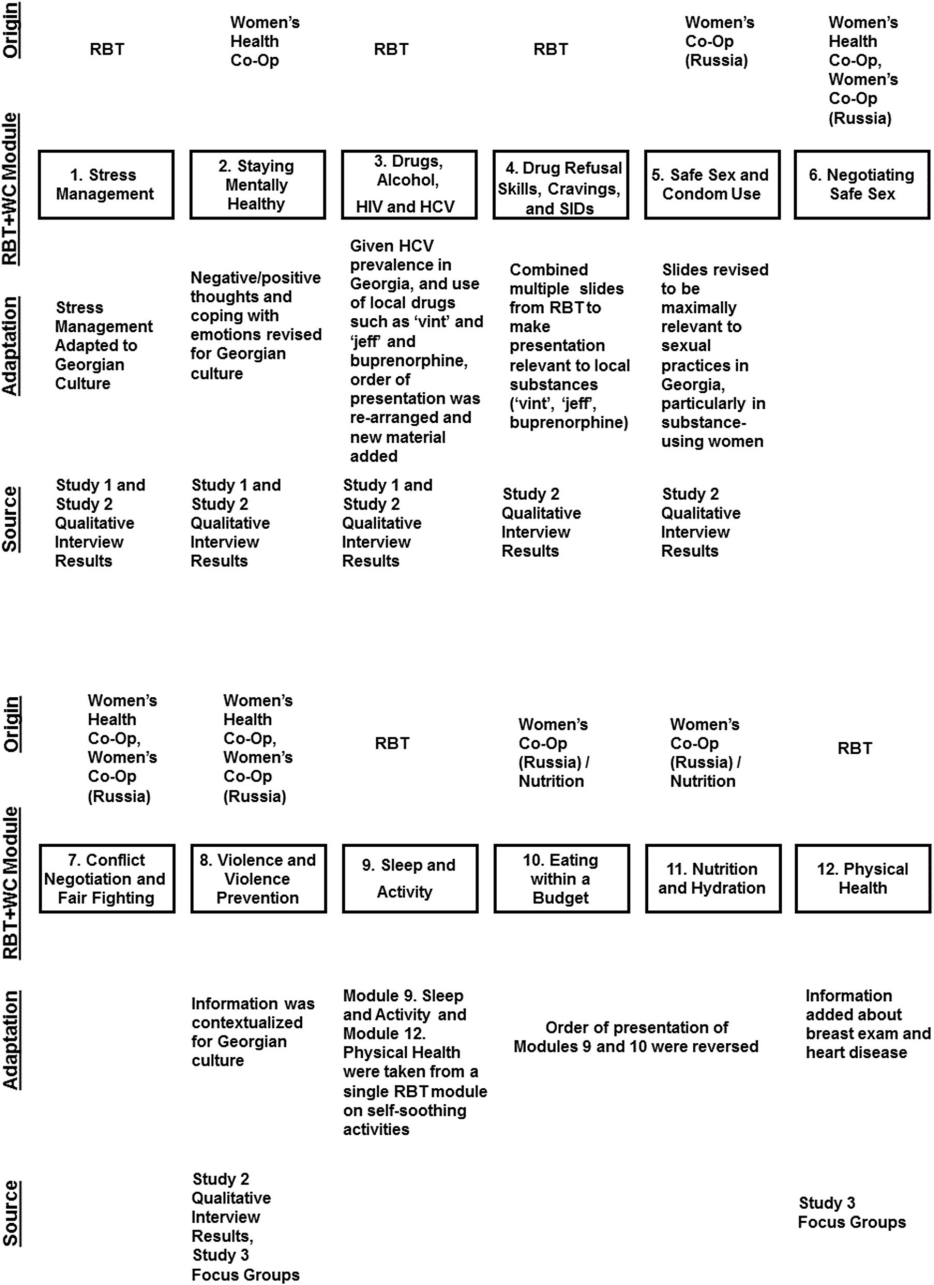
RBT + WC intervention modules: source and adaptation. Figure 2 in Jones HE, Kirtadze I, Otiashvili D, O’Grady KE, Murphy K, Zule W, Krupitsky E, Wechsberg WM. Process and product in cross-cultural treatment research: development of a culturally sensitive women-centered substance use intervention in Georgia. *J Addict* Sep 2014;Article ID 163603 © 2104 by Hendrée E. Jones et al. Used with permission

The experimental condition was developed specifically for this study, based on information gathered in the three previous IMEDI studies, Reinforcement-based Treatment plus Women’s Co-Op (RBT + WC) (see Table 1). RBT + WC represented an integration of RBT, a social-learning-theory-driven, evidence-based drug treatment intervention that employs life skills training, recreational therapy, and employment as components of a comprehensive treatment model, and WC, an intervention based in feminist theory and empowerment theory and principles of social cognitive theory. As noted in Fig. 1, two qualitative studies were conducted prior to the development of RBT + WC in order to determine the treatment needs of injection-drug-using women in Georgia, and also how to best tailor an integrated RBT and WC treatment approach consistent with Georgian culture, with a particular focus on the substance-using behaviors, sexual practices, and treatment needs of injection-drug-using women in Georgia. Moreover, feedback from our CAB, BAB, and focus groups with injection-drug-using women on the treatment manuals and intervention materials was used to further tailor the treatment to the Georgian culture, with a focus on the problems and issues of substance-using women in Georgia. In addition, as shown in Fig. 1, a pilot study was conducted in order to further revise and adapt RBT + WC, with additional feedback from our CAB, BAB, and pilot study participants. As a result of these three studies, a 12-sessions structured intervention, RBT + WC, was developed (see Fig. 2). The goals of the intervention were to reduce risky sex and substance use, and to improve physical and mental health.

**Table 1:** Theoretical Foundations of RBT and WC

RBT: Treatment Plan and Goals
Drug abstinence is the primary treatment plan focus. A Functional Assessment determines problem areas associated with drug use and is the basis for other goals. The individualized treatment plan focuses on goals directly related to decreasing/eliminating drug use. The priority of goals is dynamic, based on most pressing issues for drug abstinence initiation and continuation.
RBT: Reinforcing Small Goals to Reach the Large Goal
RBT is an active therapeutic approach. Each large treatment plan goal is broken into small steps. Progress of smaller and then larger goal behaviors are graphed at each visit (see below). Active counselor support overcomes “resistance” due to past failures.
RBT: Density of Alternative Reinforcers
RBT increases the density of alternative (non-drug) reinforcers in the person’s naturalistic environment. Thus, participants complete interest inventories and Functional Assessments with their counselor to determine what activities might serve as positive reinforcers and during periods of previous abstinence what activities or events were functioning as competing alternative reinforcers. Based upon urine tests negative for monitored substances (opioids, amphetamines, benzodiazepines, THC, buprenorphine and methadone), participants receive reward cards that have monetary value and exchangeable for goods and services.
RBT: Response to Drug Use and Proactive Outreach
Having participants provide urine samples twice weekly during treatment maximizes the ability to detect non-compliance when/if it occurs and can prevent lapses from escalating to relapses. A stimulant-positive urine test results in an individual lapse-focused counseling session (e.g., Functional Assessment; FA) and a “time out” from other RBT aspects. A missed RBT session results in proactive counseling outreach procedures that same day.
RBT: Graphing of Progress
Behaviors emitted that are congruent or incongruent with goals are graphed by the counselor with the participant. Frequent and consistent graphing of target behaviors helps to focus both counselor and participant on the tasks of treatment and also serves to provide “early warning signs” that precede a lapse or relapse. Graphing is a therapy process and does not constitute outcome measurement.
RBT: Skills Training in Recreation, Life Goals, and Other Life Skills
RBT delivers skills training in an individual or group format, with similar efficacy. Skills-training takes the form of recreational activity sampling, Social Club, and 12 educational modules (each topic is repeated three times during the 12 weeks). Each skill element is manualized, an approach previously found to be acceptable to participants.
WC: Reductions in Sex Risk and Interpersonal Violence
Four modules from Women’s Co-op were incorporated in RBT + WC. Module 1 educates women about the risks involved in alcohol and drug abuse and how certain sex behaviors increase HIV risk. Module 2 was adapted to focus on the context of sexual risk for women in Georgia, and was revised to include information gained in studies 1 and 2 (e.g., stigma, double-standards for men and women in number of sexual partners). During this time, participants are asked to practice the mechanics of correct use of male and female condoms using penile and vaginal models. Each woman has her own model to work with and has an opportunity to take home male and female condoms and experience them and return the next session to discuss how it felt to insert a female condom if they had never seen or used one. Module 3 teaches participants negotiation skills to be used with male partners and role-playing and rehearsal for practice. It directly addresses fears about intimate partner violence related to forced and unsafe sex practices and sexual negotiation. Module 4 focuses on interpersonal violence prevention, including domestic violence and rape, and strategies for violence prevention. Nonviolent resolutions are presented including a process with steps for “fair fighting” to address conflict resolution. Because the Women’s Co-op modules were a key new component of RBT + WC, it was imperative that their messages were interwoven into RBT rather than having the modules seen as independent, parallel or add-on. Thus, to integrate this effective HIV prevention into RBT, we reinforced the Women’s Co-op messages in the individual counseling sessions by graphing the frequency of safe and unsafe sex acts, discussing condom use and condom protection negotiations, and employing a functional analysis when unprotected sexual acts were reported.

*Note.* Appendix 1 in Jones HE, Kirtadze I, Otiashvili D, O’Grady KE, Murphy K, Zule W, Krupitsky E, Wechsberg WM. Process and product in cross-cultural treatment research: development of a culturally sensitive women-centered substance use intervention in Georgia. *J Addict* Sep 2014;Article ID 163603 © 2104 by Hendrée E. Jones et al. Used with permission

The control intervention was case management (CM). However, because there were no case management services available in Georgia for substance-using women, it was necessary to develop a case management intervention. The CM intervention that was developed followed the general thrust of case management offered in the US. CM participants received 12 sessions of case management and informational brochures that focused on the same issues on which RBT + WC focused.

More complete details about both interventions can be found in Jones et al. [15]

### Assessment schedule

Sexual activity of the participants were measured at baseline (study entry), post-treatment (end of the scheduled treatment, regardless of whether or not the participant completed treatment), and 3-month following treatment entry (whether or not the participant completed treatment)..

Women who completed treatment were assessed immediately at the end of treatment. Women who dropped out of treatment before the 12^th^ session were contacted and asked to return to the study site to complete their assessment. All women were contacted prior to their 3-month follow-up date and reminded to return to the study site for their follow-up assessment. Participants who failed to show for their scheduled post-treatment or follow-up appointments were contacted and their appointment re-scheduled.

### Measures

Outcomes were derived from the Revised Risk Behavior Assessment (RRBA) that had been developed for research in Russia [12]. The RRBA has 10 sections with questions about demographics and social characteristics, physical and mental health, health knowledge, alcohol and substance use, risky substance and sex risk practices, power and empowerment, conflict and victimization.

The primary sex-risk outcome was measured by the RRBA question, “Of those times you had sex with your main sex partner in the past 30 days, how many times did you have unprotected sex (e.g., without a condom)?”. A second RRBA no-yes (0:1 scored) item, “The last time you had sex with your main partner did you use a male condom?” was used to measure one of the three secondary sex-risk outcomes. The two other secondary sex-risk outcomes were derived from 11 no-yes (0:1 scored) items on the RRBA in response to the stem “In the past month did you:”. The sum of 3 items (“Ask your main sexual partner to use a condom?”, “Use a condom with your main sexual partner even if you were high on drugs?”, and “Refuse to have sex if your main sexual partner wouldn’t wear a condom?”) was used to measure Condom Use actions, and the sum of 8 items (“Ask your main sexual partner how many sex partners he has had?”, “Ask your main sexual partner if he ever injected drugs?”; “Ask your main sexual partner if he has ever shared injections?”; “Ask your main sexual partner if he has ever had an STI?”; “Ask your main sexual partner if he has HIV?”; “Ask that your main sexual partner get tested for HIV?”; “Ask that your main sexual partner get tested for STIs?”; and “Ask your main sexual partner if he has had sex with someone else?”) was used to measure Safer Sex actions.

### Statistical analysis

The design of the study was a 2 (Treatment Condition: RBT + WC v. CM) X 3 (assessment Time point: baseline v. post-treatment v. 3-month follow-up) factorial. Effects of interest were the main effects of Treatment Condition and Time, and their interaction. Outcome measures were of three types: a discrete variable in the frequency domain (frequency of unprotected sex in the past 30 days), assumed to follow a Poisson distribution; a binary variable of status of protected sex at the last sexual encounter, assumed to follow a binomial distribution; and the Condom Use actions and Safer Sex actions continuous measures, each assumed to follow a normal distribution. In the case of frequency of unprotected sex in the past 30 days, frequency of sexual intercourse in the past 30 days served as a covariate. Because all outcomes were measured repeatedly, all effects were tested using a Generalized Estimating Equations (GEE) approach. Tests of all effects were score *χ*^2^ tests. All analyses were conducted with SAS 9.3 [32]. All GEE analyses used all data that were available from participants for a given outcome.

### Power

The set correlation method [33] was used to determine the minimum effect size in the population necessary to detect an effect 80 % of the time with α = .05 for a sample size of 128 participants. The resulting effect sizes were f^2^ = .048 for the Treatment Condition main effect and f^2^ = .065 for the Time main effect and the Treatment Condition X Time interaction effect. The Time main effect and the Treatment Condition X Time interaction effect share the same effect size because they have the same degrees of freedom. These effect sizes fall in the small-to-medium range, with f^2^ = .02 considered a “small” effect and f^2^ = .15 a “medium” effect [33]. In other words, and imprecisely, under the assumption that the effect in the population was ≥ .048 or ≥ .065, for the Treatment Condition, and the Time and Treatment Condition X Time effects, respectively, there is, in the long run, an 80 % chance of concluding that the respective effect is significant if α was set to .05 and data were provided by 128 participants.

## Results

### Participants

The number of women screened was 173, of whom 128 met eligibility criteria. The 45 ineligible women screened out of the study because of: no drug use (*n* = 5), non-verifiable injection-drug-use status (*n* = 25), not sexually active in the past 30 days (*n* = 4), and no injection drug use and not sexually active (*n* = 11). Of the 128 women who entered the trial, 114 (89 %) had injected heroin, 104 (81 %) had injected buprenorphine, 96 (75 %) had injected the homemade amphetamine-type stimulants ‘Vint’ or ‘Jeff’, and 78 (61 %) had injected the home-made opioid desomorphine ‘crocodile’, with first injection at 26.3 years of age (*SD* = 8.1), with a friend as the person most likely to have helped them first inject (*n* = 75, 59 %). All women had a history of injection drug use, 43 (34 %) had a history of needle sharing, 3 (2 %) were HIV-positive by Western blot testing at study entry, 66 (52 %) were HCV-positive by Eliza testing at study entry, and they had a mean number of days of opioid use in the past 30 days of 10.9 (*SD* = 10.8). Of the 128 women who entered the trial, 113 (88 %) completed treatment, 112 (88 %) were assessed at post-treatment, and 113 (88 %) were assessed at 3-month follow-up.

Table 2 presents demographic and background information on the 128 participants, while Table 3 presents information regarding their sexual history. Participants were predominantly Georgian, fairly well educated, largely unemployed, generally unmarried but in a long-term sexual relationship. They typically engaged in sexual intercourse for the first time in their teen years [*M* = 18.2 (*SD* = 3.3), 73 % at 19 years of age or younger, range: 12–30]. Most women used condoms infrequently, although almost 20 % worried about the sexual fidelity of their partner and almost 20 % reported having had sex with what in Georgia is known as a “secret sex partner”, someone other than their main sex partner, and without his knowledge. Many of them reported using drugs before or during their last sexual contact. Many participants reported that their main sex partner was often verbally abusive, but their partners were infrequently physically abusive. Finally, they were generally knowledgeable about HIV, with a mean score of 8.2 (*SD* = 1.7; range: 3–11) on an measure of HIV knowledge that contained 11 true-false questions: "People with HIV always look skinny and sick" (15 % incorrect); "An HIV positive woman can pass the virus to her baby before it is born" 22 % incorrect); "Babies can get HIV/AIDS through breast feeding from their HIV-infected mothers" (26 % incorrect); "A couple who both have HIV do not need to use condoms" (50 % incorrect); "People with HIV/AIDS are more likely to get TB (bad cough, shortness of breath, night sweats)" (18 % incorrect); "There are medicines that a pregnant HIV positive woman can take to keep from giving HIV to her baby" (38 % incorrect); "Blood (including menstrual) can transmit HIV to someone else" (4 % incorrect); "Sperm and female discharge can transmit HIV to someone else" (4 % incorrect); "Spit can transmit HIV to someone else" (49 % incorrect); "Injecting drug users can transmit HIV by sharing needles" (4 % incorrect); "Antiretrovirals (ARV) and other drugs to treat AIDS can protect a baby from HIV" (41 % incorrect).

**Table 2:** Baseline Demographic and Background Variables for the Study Entry Sample, the Samples that Completed and Failed to Complete Treatment, and the Reinforcement-based Treatment and Case Management Samples at Baseline (*N* = 128)

	Study Entry Sample (*N* = 128)	Completed Treatment Sample (*n* = 113)	Failed to Complete Treatment Sample (*n* = 15)	Tests of Treatment Completion Status	Reinforcement- based Treatment Sample (*n* = 64)	Case Management Sample (*n* = 64)	Tests of Treatment Condition
Variable	*M* (*SD*) or *n* (%)			*p*	*M* (*SD*) or *n* (%)		*p*
Age	41.2 (10.0)	41.8 (10.1)	36.9 (8.6)	0.07	42.0 (10.1)	40.4 (10.0)	0.36
National Origin:				--			0.78
Georgian	114 (89.1 %)	99 (87.6 %)	15 (100 %)		56 (87.5 %)	58 (90.6 %)	
Russian	5 (3.9 %)	5 (4.4 %)	0		3 (4.7 %)	0	
Armenian	1 (0.8 %)	1 (0.9 %)	0		1 (1.6 %)	2 (3.1 %)	
Other	8 (6.3 %)	8 (7.1 %)	0		4 (6.3 %)	4 (6.3 %)	
Education:				--			0.48
Incomplete school	8 (6.3 %)	8 (7.1 %)	1 (6.3 %)		4 (6.3 %)	4 (6.3 %)	
Completed school	36 (28.1 %)	31 (27.4 %)	5 (33.3 %)		20 (31.3 %)	16 (25.0 %)	
Current student	4 (3.2 %)	4 (3.5 %)	0		2 (3.1 %)	2 (3.1 %)	
Incomplete university	11 (8.6 %)	9 (8.0 %)	2 (13.3 %)		3 (4.7 %)	8 (12.5 %)	
Completed university	53 (41.4 %)	48 (42.5 %)	5 (33.3 %)		27 (42.1 %)	26 (40.7 %)	
Incomplete further degree	1 (0.8 %)	0	1 (6.7 %)		1 (1.6 %)	0	
Completed further degree	13 (10.2 %)	11 (9.7 %)	2 (13.3 %)		6 (9.4 %)	7 (10.9 %)	
Other	2 (1.6 %)	2 (1.8 %)	0		1 (1.6 %)	1 (1.6 %)	
Employment status:				0.74			1
Full-time	6 (4.7 %)	6 (5.3 %)	0		3 (4.7 %)	3 (4.7 %)	
Part-time	7 (5.5 %)	7 (6.2 %)	0		4 (6.3 %)	3 (4.7 %)	
Part-time, self-employed	7 (5.5 %)	5 (4.45 %)	2 (13.3 %)		2 (3.1 %)	5 (7.8 %)	
Student	1 (0.8 %)	1 (0.9 %)	13 (86.7 %)		1 (1.6 %)	0	
Other	2 (1.6 %)	2 (1.8 %)	0		2 (3.1 %)	0	
Unemployed	105 (82.0 %)	92 (81.4 %)			52 (81.3 %)	53 (82.8 %)	
Marital status:				--			0.74
Married to main sexual partner	38 (29.7 %)	36 (31.9 %)	2 (13.3 %)		21 (32.8 %)	17 (26.6 %)	
Living with main sexual partner	30 (23.4 %)	28 (24.8 %)	2 (13.3 %)		15 (23.4 %)	15 (23.4 %)	
Not living with main sexual partner	56 (43.8 %)	46 (40.8 %)	10 (66.7 %)		26 (40.6 %)	30 (46.9 %)	
No main sexual partner	4 (3.1 %)	3 (2.7 %)	1 (6.7 %)		2 (3.1 %)	2 (3.1 %)	
Number of years been together with main sex partner (*n* = 124)	9.4 (10.6)	10.0 (10.8)	5.2 (7.4)	0.09	10.5 (11.7)	8.3 (9.1)	0.27
Living with anyone who uses drugs: Yes	52 (40.6 %)	47 (41.6 %)	5 (33.3 %)	0.59	21 (32.8 %)	31 (48.4 %)	0.11
Number of times main sex partner used drugs in the past 30 days (*n* = 122)	8.3 (9.8)	8.3 (9.8)	8.6 (9.7)	0.92	7.3 (10.9)	9.3 (9.0)	0.25
How many friends would you be able to ask for help	2.8 (2.6)	2.7 (2.6)	3.8 (2.9)	0.15	3.0 (2.5)	2.7 (2.8)	0.49
How many of these friends currently use drugs or alcohol	1.2 (2.2)	1.1 (2.1)	1.9 (2.9)	0.15	1.2 (2.0)	1.2 (2.4)	0.87

Notes. Likelihood ratio exact tests were used for categorical variables, and *t* tests for continuous variables. Percentages represent column percentages for the respective variable. Percentages do not total to 100 % due to rounding. – indicates a test was not conducted because a cell(s) had expected count(s) less than 5, and as such an asymptotic likelihood ratio exact test might not yield a valid test of significance. Tests for national origin were between Georgian and non-Georgian groups (collapsing the Russian, Armenian, and Other groups into a non-Georgian group). Tests for education status were between completed school, incomplete university/technical study, completed university/technical study, and further degree groups (ignoring the *n* = 6 participants in the incomplete school, student and other groups). Tests for employment status were between unemployed and other groups (collapsing the employed full-time, employed part-time, self-employed part-time, student and others into the other group). Test for marital status were between the married to main sexual partner, living with main sexual partner, and having a main sexual partner not living with him groups (ignoring the *n* = 4 participants with no main sexual partner). Missing data were as follows: 4 participants with no main sexual partner, leading to missing information on questions regarding number of years with main sexual partner and number of times main sexual partner used drugs in the past 30 days, which had 2 additional cases of non-response, and as such – in the Completed Treatment sample, 4 participants were missing data on number of years with main sexual partner and 5 participants on number of times main sexual partner used drugs in the past 30 days and in the Failed to Complete Treatment sample, 1 participant was missing information on number of times main sexual partner used drugs in the past 30 days; and in the RBT Condition, 2 participants were missing data on number of years with main sexual partner and 4 participants on number of times main sexual partner used drugs in the past 30 days, while in the Usual Care Condition, 2 participants each were missing data on number of years with main sexual partner and on number of times main sexual partner used drugs in the past 30 days, respectively

**Table 3:** Baseline Sexual History for the Study Entry Sample, the Samples that Completed and Failed to Complete Treatment, and the Reinforcement-based Treatment and Usual Care Samples at Baseline (*N* = 128)

	Study Entry Sample (*N* = 128)	Completed Treatment Sample (*n* = 113)	Failed to Complete Treatment Sample (*n* = 15)	Tests of Treatment Completion Status	Reinforcement-based Treatment Sample (*n* = 64)	Case Management Sample (*n* = 64)	Tests of Treatment Condition
Variable	*M (SD) or n (%)*			*p*	*M (SD) or n (%)*		*p*
Age at first vaginal sex	18.2 (3.3)	18.3 (3.6)	17.7 (3.3)	0.55	18.1 (3.3)	18.4 (3.4)	0.62
Person with whom first vaginal sex was experienced:				--			0.67
Husband	96 (75 %)	85 (75.2 %)	11 (73.3 %)		48 (75.0 %)	48 (75.0 %)	
Boyfriend	27 (21.1 %)	23 (20.4 %)	4 (26.7 %)		12 (18.8 %)	15 (23.4 %)	
Stranger	1 (0.8 %)	1 (0.9 %)	0		0	1 (1.6 %)	
Friend of family	1 (0.8 %)	1 (0.9 %)	0		1 (1.6 %)	0	
Other	3 (2.3 %)	3 (2.7 %)	0		3 (4.7 %)	0	
More than 1 sex partner in the past 30 days: yes	7 (5.5 %)	6 (5.3 %)	1 (6.7 %)	--	5 (7.8 %)	2 (3.1 %)	--
Number of times had sexual intercourse in the past 30 days	11.6 (11.0)	12.1 (11.5)	8.9 (5.9)	0.3	11.5 (13.9)	11.7 (7.3)	0.92
Number of times of unprotected sexual intercourse in the past 30 days	9.9 (11.7)	10.5 (12.1)	5.9 (7.6)	0.15	10.2 (14.2)	9.7 (8.6)	0.82
Condom use at last sexual intercourse with main partner: yes	25 (20.2 %)	18 (16.4 %)	7 (50.0 %)	--	11 (17.7 %)	14 (22.6 %)	0.66
Main sex partner is having sex with someone else:				1			0.01
Definitely yes	10 (8.1 %)	9 (8.2 %)	1 (7.1 %)		9 (14.5 %)	1 (1.6 %)	
Probably yes	13 (10.5 %)	11 (10.0 %)	2 (14.3 %)		7 (11.3 %)	6 (9.7 %)	
Probably not	19 (15.3 %)	18 (16.4 %)	1 (7.1 %)		5 (8.1 %)	14 (22.6 %)	
Definitely not	82 (66.1 %)	72 (65.4 %)	10 (71.4 %)		41 (66.1 %)	41 (66.1 %)	
Lifetime ever sexual intercourse with a secret sex partner: yes	25 (19.5 %)	21 (18.6 %)	4 (26.7 %)	0.74	13 (20.3 %)	12 (18.8 %)	1
Last sex was with main sex partner: yes	119 (93.0 %)	106 (93.8 %)	13 (86.7 %)	--	58 (90.6 %)	61 (95.3 %)	0.49
Drug use just before or during last sex: yes	98 (76.6 %)	86 (76.1 %)	12 (80.0 %)	--	46 (71.9 %)	52 (81.3 %)	0.3
Partner drug use just before or during last sex: yes	65 (50.8 %)	59 (52.2 %)	6 (40.0 %)	0.42	29 (45.3 %)	36 (56.3 %)	0.29
Unprotected sex at last sexual contact: yes	101 (78.9 %)	93 (82.3 %)	8 (53.3 %)	--	52 (81.3 %)	49 (76.6 %)	0.67
HIV Knowledge	8.2 (1.7)	8.3 (1.7)	7.9 (1.4)	0.42	8.1 (1.7)	8.3 (1.6)	0.61
In the past 30 day did your main sex partner:							
insult you or make you feel bad about yourself?	32 (25.8 %)	28 (25.5 %)	4 (28.6 %)	--	16 (25.8 %)	16 (25.8 %)	1
belittle or humiliate you in front of other people?	10 (8.1 %)	8 (7.3 %)	2 (14.3 %) + D27	--	6 (9.7 %)	4 (6.5 %)	0.74
do things to scare or intimidate you on purpose for example by the way he looked at you, by yelling and smashing things?	6 (4.8 %)	6 (5.5 %)	0	--	4 (6.5 %)	2 (3.2 %)	--
threaten or hurt you?	3 (2.4 %)	3 (2.7 %)	0	--	3 (4.8 %)	0	
stopped from seeing any of your friends?	0	0	0		0	0	
slap you or throw something at you that could have hurt you?	5 (4.0 %)	5 (4.6 %)	0	--	3 (4.8 %)	2 (3.2 %)	--
hit you with a fist or with something else which could hurt you?	0	0	0		0	0	
push or shove you?	1 (0.8 %)	1 (0.9 %)	0	--	0	1 (1.6 %)	--
kick, drag, beat, choke or burn you?	0	0	0		0	0	
threaten to use or actually use a gun, knife or other weapon against you?	0	0	0		0	0	
physically force you to have sex when you did not want to?	1 (0.8 %)	1 (0.9 %)	0	--	1 (1.6 %)	0	--
did you have sex with your main sexual partner when you did not want to because you were afraid of what he might do?	1 (0.8 %)	1 (0.9 %)	0	--	1 (1.6 %)	0	--
force you to have anal (i.e., bum) sex	0	0	0		0	0	

Notes. Likelihood ratio exact tests were used for categorical variables, and *t* tests for continuous variables. Percentages represent column percentages for the respective variable. Percentages do not total to 100 % due to rounding. – indicates a test was not conducted because a cell(s) had expected count(s) less than 5, and as such an asymptotic likelihood ratio exact test might not yield a valid test of significance. Missing data were as follows: 4 participants had no main sexual partner (see Table 2), leading to missing information on the questions regarding condom use at last sexual intercourse with main partner, main sex partner is having sex with someone else, and in the past 30 day did your main sex partner, and as such 3 participants in the Completed Treatment sample and 1 participant in the Failed to Complete Treatment sample were missing data on these variables; and 2 participants each in the RBT and Usual Care Conditions were missing data on these variables

### Psychometric characteristics of the condom use and safer Sex measures

Both these measures showed excellent internal consistency reliability (at baseline, treatment completion, and 3-month follow-up: αs = .84, .86, and .92; and .87, .89, and .91, respectively), although the means of both measures were low [at baseline, treatment completion, and 3-month follow-up: Condom Use *M* (*SD*; range) = .6 (1.0; 0–3), .6 (1.1; 0–3), and .4 (1.0; 0–3); Safer Sex *M* (*SD*; range) = 1.2 (2.0; 0–8), .8 (1.8; 0–8), and 1.0 (2.1; 0–8), respectively].

### Results of inferential analyses

Tables 4 and 5 detail the GEE results. Analyses revealed two significant findings: (1) A main effect for Treatment Condition, with CM having significantly overall higher Safer Sex actions [*M* = 1.2 (*SE* = 0.2)] than RBT + WC [*M* = 0.7 (*SE* = 0.2)], *χ*^2^(1) = 4.83, *p* = .03, likely due to the fact that the CM mean is significantly [*χ*^2^(1) = 4.32, *p* < .04] higher than the RBT + WC mean at baseline, and non-significantly higher at post-treatment and 3-month follow-up; and, (2) a significant Time main effect, with a decrease over Time for past 30-day number of unprotected sex acts [*M* = 8.5 (*SE* = 0.6) at baseline, M = 6.1 (*SE* = 0.5) at post-treatment, and *M* = 6.6 (*SE* = 0.4) at 3-month follow-up], *χ*^2^(2) = 31.21, *p* < .001. Post hoc tests of the Time main effect means revealed a significant decline of more than 20 % in the number of past-30-day unprotected sex acts from baseline to post-treatment, *χ*^2^(1) = 16.16, *p* < .001, and from baseline to 3-month follow-up, *χ*^2^(1) = 9.61, *p <* .002. The post-treatment mean and 3-month follow-up mean were not significantly different from each other, *χ*^2^(1) = 0.79, *p* = .37. Unprotected sex at the last encounter and Condom Use actions were nonsignificant (*p*s > .15 for all effects), with the predicted probability of unprotected sex at the last encounter in the total sample equal to .77 (*SE* = .19). Table 4 repeats all tests of significance for Treatment Condition, Time, and Treatment Condition X Time reported in this paragraph, as well as the Treatment Condition, Time, and Treatment Condition X Time test statistics and *p*-values for all nonsignificant effects for the primary outcome variables, while Table 5 repeats the means and standard errors reported here, as well as for all nonsignificant Treatment Condition, Time, and Treatment Condition X Time effects for the primary outcome variables.

**Table 4:** Results of Inferential Analyses for Primary and Secondary Sex-Risk Outcome Measures (*N* = 128)

	Treatment Condition		Time		Treatment Condition X Time	
	*χ* ^2^	*p*	*χ* ^2^	*p*	*χ* ^2^	*p*
Primary Outcomes						
Past-30-day frequency of unprotected sexual intercourse	1.64	0.2	31.21	<.001	1.47	0.48
Secondary Outcomes						
Unprotected sex at the last encounter: yes	0.03	0.87	2.78	0.25	0.79	0.68
Condom Use Actions	0.02	0.89	2.71	0.26	3.76	0.15
Safer-sex Actions	4.83	0.03	2.29	0.32	1.81	0.4

*Notes. N* = 124 (128–4 with no main sex partner) at baseline; for secondary outcomes, *N* = 96 (128–16 with no main sex partner and 16 failed to return for assessment) at post-treatment assessment, and *N* = 86 (128–27 with no main sex partner and 15 failed to return for assessment) at 3-month follow-up assessment, while for the primary outcome, *N* = 92 (128–16 with no main sex partner and 16 failed to return for assessment plus an additional 4 participants who had a main sex partner who declined to answer these two questions) at post-treatment assessment and *N* = 74 (128–27 with no main sex partner and 15 failed to return for assessment plus an additional 12 participants who had a main sex partner who declined to answer these two questions). *df* = 1 for Treatment Condition, *df* = 2 for Session, and *df* = 2 for Treatment Condition X Session. Past-30-day number of times engaging in unprotected intercourse was a count variable assumed to follow a Poisson distribution, unprotected sex at the last encounter was a binary variable assumed to follow a binomial distribution, and condom self-efficacy and safe-sex self efficacy were continuous variables assumed to follow a normal distribution

**Table 5:** Means (Standard Errors) from Inferential Analyses for Primary and Secondary Sex-Risk Outcome Measures (*N* = 128)

	Treatment Condition		Time			Treatment Condition X Time					
						RBT			Usual Care		
	RBT	Uusal Care	Baseline	Post-Treatment	3-month Follow-up	Baseline	Post-Treatment	3-month Follow-up	Baseline	Post-Treatment	3-month Follow-up
Primary Outcomes											
Past-30-day frequency of unprotected sexual intercourse	6.6 (0.5)	7.5 (0.5)	8.5 (0.6)	6.1 (0.5)	6.6 (0.4)	7.8 (0.7)	5.6 (0.6)	6.5 (0.7)	9.2 (0.8)	6.8 (0.6)	5.7 (0.6)
Secondary Outcomes											
Unprotected sex at the last encounter: yes	0.8 (0.05)	0.8 (0.04)	0.8 (0.04)	0.7 (0.04)	0.8 (0.04)	0.8 (0.05)	0.7 (0.04)	0.8 (0.06)	0.8 (0.05)	0.7 (0.06)	0.8 (0.06)
Condom Use Actions	0.5 (0.1)	0.6 (0.1)	0.6 (0.1)	0.6 (0.1)	0.4 (0.1)	0.5 (0.1)	0.7 (0.2)	0.4 (0.1)	0.6 (0.1)	0.5 (0.1)	0.5 (0.2)
Safer-sex Actions	0.7 (0.2)	1.2 (0.2)	1.1 (0.2)	0.8 (0.2)	0.9 (0.2)	0.7 (0.2)	0.6 (0.2)	0.6 (0.3)	1.4 (0.3)	0.9 (0.2)	1.3 (0.3)

*Notes.* RBT = Reinforcement Based Treatment. *N* = 124 (128–4 with no main sex partner) at baseline; for secondary outcomes, *N* = 96 (128–16 with no main sex partner and 16 failed to return for assessment) at post-treatment assessment, and *N* = 86 (128–27 with no main sex partner and 15 failed to return for assessment) at 3-month follow-up assessment, while for the primary outcome, *N* = 92 (128–16 with no main sex partner and 16 failed to return for assessment plus an additional 4 participants who had a main sex partner who declined to answer these two questions) at post-treatment assessment and *N* = 74 (128–27 with no main sex partner and 15 failed to return for assessment plus an additional 12 participants who had a main sex partner who declined to answer these two questions). Model-estimated means for the Poisson variable (past-30-day frequency of unprotected sexual intercourse) have been back-transformed into the metric of the original variables. Model-estimated means for the binomial variable (unprotected sex at the last encounter) are the predicted probabilities

## Discussion

### Participants

Most of the women in this study reported only engaging in sex with their main partner, and they reported using condoms infrequently. Overall, 75 % of the sample first became sexually active with their husbands. Only 20 % reported a secret (i.e., an extramarital/outside) sex partner, and only 7 % reported that their most recent intercourse was with someone other than their main sex partner. Moreover, 77 % of participants reported using substances before or during their last sexual intercourse, and they reported that 51 % of their partners had used substances before or during intercourse. Under these circumstances, it is not surprising that 79 % reported not using a condom the last time they had sex.

Although women reported that their main sex partners were often verbally abusive, they also reported that their main sex partners rarely were physically intimidating or abusive to them. This relatively low level of exposure to violence is decidedly different than samples of substance-using women in the US, where rates of physical and sexual violence exceed 80 % [34].

### Feasibility of RBT + WC

Findings would suggest that implementation of culturally sensitive, women-focused treatment in Georgia can be successfully undertaken. We were able to screen 173 women using venue-sampling methods, and recruit 128 women during a 12-month recruitment period, from 1 April 2013 to 15 April 2014. Moreover, 113/128 (88 %) completed treatment, and 112 (88 %) and 113 (88 %) provided post-treatment and 3-month follow-up data, respectively. Although the trial was relatively short-term in its focus on outcomes, these percentages are excellent, given drop-out rates for outpatient substance use treatment have been estimated at 23-50 % [35].

We have discussed one of the striking cultural differences between research in the US and research in Georgia in a previous publication, [15] where we noted that research is a novel experience to the Georgian populace, and so Georgian women whom we have recruited have often been quite interested in helping our efforts to conduct our research and collect data. This phenomenon may have in part contributed to our success in both recruitment into the study and retention of participants, and collection of follow-up data.

### Efficacy of RBT + WC

Results did not support the efficacy of RBT + WC relative to case management in regard to risky sexual behavior. The fact that the CM condition had higher scores on the Safer Sex measure than did the RBT + WC condition is likely due to pretreatment differences between the two conditions – the CM Safer Sex means are higher than the RBT + WC means at all three assessments. Moreover, there was only one significant result indicating change over time in participant’s sex-risk behavior, for number of unprotected sex acts during the preceding 30-day period. Taken together, these findings suggest that the participants did not change their risky sexual behavior during the course of treatment, irrespective of whether it was RBT + WC or case management, with the possible exception of the finding that weakly suggests that RBT + WC and CM were equally effective in producing change in risky sexual behavior over the course of the study – or, alternatively, that participants might have somehow been sensitized to the need to use condoms due to participation in the study. It is particularly distressing that the Condom Use and Safer Sex means in the total sample were quite low throughout the study. However, it is possible than many of the participants were in longer-term relationships and had already learned the injection drug use and HIV status of their partners, and so did not believe it necessary to use a condom for STI protection. We did not directly assess trust in the relationship. We do note that although the predicted probability of unprotected sex at the last sexual encounter in the total sample was .77, while, as Table 3 notes, the percentage of participants who indicated that their main sexual partner was “probably not” having sex with someone else was 15 % and “definitely not” having sex with someone else was 66 %, a combined percentage of 81 %, the relationship between these two variables was nonsignificant.

It is also the case that the majority women in Georgia (58 %) have historically never utilized any method of contraception, with the estimate in 2005 of women not currently using any contraceptive method of 72 % [36]. Moreover, the use of condoms in 2005 among Georgian women was estimated at 9 % [37]. Thus, historical forces, together with a strong Georgian Orthodox opposition to the use of birth control methods, may have also operated to blunt the impact of our intervention – although it was the case that the women as a group did engage in unprotected sexual intercourse less frequently at the conclusion of the study and at 3-month follow-up that at baseline assessment, suggesting the possibility of some impact of exposure to information about condom use that was available in both treatment conditions.

In considering future research examining the efficacy of RBT + WC, the open question is whether RBT + WC could be effective with a different population of injection-drug-using women in Georgia, and/or whether RBT + WC needs to be further tailored to increase its impact on injection-drug-using women in Georgia. Although our goal was to sample broadly among injection-drug-using women in Georgia, our sample was largely composed of injection-drug-using women who were in relatively stable relationships with a single male sex partner. Thus, the goal of RBT + WC to increase condom use could have been blunted by the fact that the sample as a whole felt little need to change their condom use practices, given long-term and generally exclusive heterosexual relationships. We need to determine whether the sample we recruited represents the dominant percentage of injection-drug-using women in Georgia, or whether this population has, as we had expected, a large percentage of women who have no stable heterosexual partner. If the former circumstance is true, then we would need to further adapt RBT + WC so it addressed the needs of injection-drug-using women with a stable heterosexual sex partner. Such an intervention could be tailored along the lines of the couple-based intervention developed by El-Bassel, Gilbert, and colleagues [38–42]. Tailoring could be based in part on our own research with treating female partners of opioid-injecting men in Georgia [43]. If the later circumstance is true, we would want to further explore the efficacy of the current version of RBT + WC.

### Limitations

There were several limitations to the present study. First, the recruitment strategy may or may not have recruited a sample representative of women in Georgia who injected substances. However, there are so few women who inject drugs in treatment in Georgia [21] that it would not be possible to recruit a sample from women seeking treatment for their drug use – although it is likely that this sample would be representative of women in Georgia who inject drugs. Nonetheless, the recruitment strategy and our resulting sample limits our ability to generalize our findings to women who inject drugs in Georgia. Second, the sample size was relatively small, as one of the primary goals of the study was to assess the feasibility of the intervention. Accordingly, we had limited power to detect small intervention effects. Third, it is possible that the active ingredients in the case management intervention developed as the control condition specifically for this study were sufficiently impactful that differences between the RBT + WC condition were minimized, adversely affecting the ability to detect an effect. However, not offering some form of treatment to the control condition participants would have been unethical. Finally, despite the fact that the RBT + WC intervention was developed on the basis of data collected in three previous studies with the goal of meeting the needs of injection-drug-using women in Georgia, it is possible that the RBT + WC intervention was not sufficiently tailored to their needs.

### Implications for prevention and policy

Unsurprisingly, the main implication for prevention is that there is strong need for changes in both public policy and public health that recognize the need for condom use. In some ways, injection-drug-using women would seem to recognize the greater need for the use of condoms than occurs in the general Georgian female population, given the fact the percentage in the general population was less than 10 % in 2005. However, that an estimated 77 % of a high-risk population did not use a condom at their most recent sexual encounter is alarming. Yet public policy and public health efforts to encourage condom use are at odds with the position of the Georgian Orthodox Church regarding contraception, which it condemns. The issue is further compounded by the fact that, although the rate of HIV infection is steadily rising in Georgia, the absolute number of cases remains low, [26] meaning that public attention has largely not focused on HIV infection as a problem facing general society. The problem is then further compounded by the fact that injection-drug-using women are highly stigmatized in Georgian culture, extending even to health service providers [20]. Otiashvili and colleagues [44] have identified four areas of concern in developing successful treatment programs in Georgia for injection-drug-using women: sociocultural issues; policy issues; programmatic/structural issues; and personal/Interpersonal Issues, and have suggested that solutions must be sought in four areas: public health campaigns; development and implementation of comprehensive women-specific confidential treatment models; policy reform; and empowering women. More complete details of needed reforms to the service delivery system in Georgia can be found in their article.

## Conclusions

Women who inject drugs in Georgia are engaging in risky sexual practices, and are in need of an intervention that addresses these risky behaviors. Neither a comprehensive women-centered intervention based on the twin platforms of RBT and WC – two interventions that have been successfully adapted to treatment in international settings – nor case management tailored to the needs of women who inject drugs in Georgia was effective in changing risky sexual behaviors. Whether the sample size was insufficient to detect an effect, whether the effect size was smaller than anticipated, and/or whether the sample did not come from a population for which the intervention would be efficacious are open questions. Our previous research has suggested the importance of social, cultural, and treatment provider barriers to treatment entry and treatment retention for injection-drug-using women in Georgia and outlined further directions for treatment [44]. Further research focusing on the circumstances under which women who inject drugs in Georgia continue to engage in risky sexual behaviors needs to be undertaken in order to revise RBT + WC so that it can lead to changes in these behaviors. In particular, it may be that RBT + WC would be effective with Georgian women without a stable sexual partner who inject drugs and engage in sexual activity. In regard to both of these missions, it might be important to ‘expand the lens’ and focus more broadly on women in the Southern Caucasus region, and determine commonalities and unique characteristics associated injection drug use by women in the region as a way to better develop a comprehensive women-centered intervention for this highly stigmatized and highly vulnerable population.
